# Perioperative outcomes of robotic and laparoscopic surgery for colorectal cancer: a propensity score-matched analysis

**DOI:** 10.1186/s12957-023-03138-y

**Published:** 2023-08-30

**Authors:** Emile Farah, Andres A. Abreu, Benjamin Rail, Javier Salgado, Georgios Karagkounis, Herbert J. Zeh, Patricio M. Polanco

**Affiliations:** Department of Surgery, Division of Surgical Oncology, University of Texas Southwestern Medical Center, 5323 Harry Hines Blvd, Dallas, TX 75390 USA

**Keywords:** Colorectal cancer, Colorectal surgery, Robotic, Laparoscopic

## Abstract

**Background:**

Robotic colorectal surgery is becoming the preferred surgical approach for colorectal cancer (CRC). It offers several technical advantages over conventional laparoscopy that could improve patient outcomes. In this retrospective cohort study, we compared robotic and laparoscopic surgery for CRC using a national cohort of patients.

**Methods:**

Using the colectomy-targeted ACS-NSQIP database (2015–2020), colorectal procedures for malignant etiologies were identified by CPT codes for right colectomy (RC), left colectomy (LC), and low anterior resection (LAR). Optimal pair matching was performed. “Textbook outcome” was defined as the absence of 30-day complications, readmission, or mortality and a length of stay < 5 days.

**Results:**

We included 53,209 out of 139,759 patients screened for eligibility. Laparoscopic-to-robotic matching of 2:1 was performed for RC and LC, and 1:1 for LAR. The largest standardized mean difference was 0.048 after matching. Robotic surgery was associated with an increased rate of textbook outcomes compared to laparoscopy in RC and LC, but not in LAR (71% vs. 64% in RC, 75% vs. 68% in LC;* p* < 0.001). Robotic LAR was associated with increased major morbidity (7.1% vs. 5.8%; *p* = 0.012). For all three procedures, the mean conversion rate of robotic surgery was lower than laparoscopy (4.3% vs. 9.2%; *p* < 0.001), while the mean operative time was higher for robotic (225 min vs. 177 min; *p* < 0.001).

**Conclusions:**

Robotic surgery for CRC offers an advantage over conventional laparoscopy by improving textbook outcomes in RC and LC. This advantage was not found in robotic LAR, which also showed an increased risk of serious complications. The associations highlighted in our study should be considered in the discussion of the surgical management of patients with colorectal cancer.

## Background

The surgical management of colorectal cancer (CRC) has evolved in the last decade with the introduction of novel surgical equipment, techniques, and the rapid expansion of robotic surgery [1]. General surgery has become the largest market for robotics with a 24-fold increase since 2010 [2]. Proponents of this new technology allege improved outcomes and safety for common procedures, such as colorectal resections. However, evidence on the benefit of adopting the robotic platform for CRC remains limited and may not reflect real-world practice. CRC remains one of the most common types of cancer and a primary contributor to the increase in cancer-related death worldwide [3]. Despite a decrease in the incidence and mortality of CRC among adults older than 50 years of age, we are observing an alarming increase in CRC among younger adults since the early 1990s [4–6]. These trends highlight the importance of optimizing surgical treatment strategies for CRC.

The national operative case log database of the ACGME for general surgery residents showed an increase in the proportion of minimally invasive surgery in colorectal cases from 8% in 2003 to 43% in 2018 [7]. This increase was accompanied by evidence supporting laparoscopic colorectal surgery as superior to open surgery, with faster recovery, less postoperative pain, shorter length hospital stay, and comparable oncologic outcomes [8–10]. Recently, robotic surgical systems were introduced to overcome certain limitations of laparoscopy by offering better 3D visualization, a stable camera, bimanual dexterity, tremor reduction, and improved ergonomics [11]. Therefore, robotic-assisted colorectal surgery has garnered wide acceptance despite the lack of convincing evidence on its advantages over laparoscopy [12–18]. Most studies addressing this comparison are based on single-institutional data, small sample size, or a heterogeneous patient cohort without appropriate control populations [19, 20].

To address this knowledge gap, we conducted a retrospective cohort study evaluating the perioperative outcomes of robotic and laparoscopic surgery for CRC in a propensity score-matched analysis. Using the colectomy-targeted American College of Surgeons-National Surgical Quality Improvement Program (ACS-NSQIP) database, we compared robotic and laparoscopic right colectomy (RC), left colectomy (LC), and low anterior resection (LAR). If robotic colorectal surgery offers an advantage over laparoscopy, we hypothesized that perioperative outcomes would be more favorable after robotic assisted surgery.

## Methods

### Data source

The ACS-NSQIP is a nationally validated, risk-adjusted, outcomes-based program used to track and refine surgical care based on 30-day patient outcomes. This program collects data on more than 250 variables, including demographics, preoperative risk factors, intraoperative variables, and 30-day postoperative morbidity and mortality. To ensure the highest quality standards, data are collected and maintained by a dedicated surgical clinical reviewer at each participating institution. The ACS-NSQIP also includes rigorous data field definitions with ongoing review, conducts frequent audits of participating sites, and requires annual certification exams for surgical clinical reviewers [21]. Using the unique “CASEID” variable, we merged the main NSQIP to the colectomy-targeted participant user data file containing 23 additional variables specific to colorectal operations. This study was reviewed by the University of Texas Southwestern Human Research Protection Program and deemed exempt from IRB approval or oversight.

### Study design and population

**Fig. 1:**
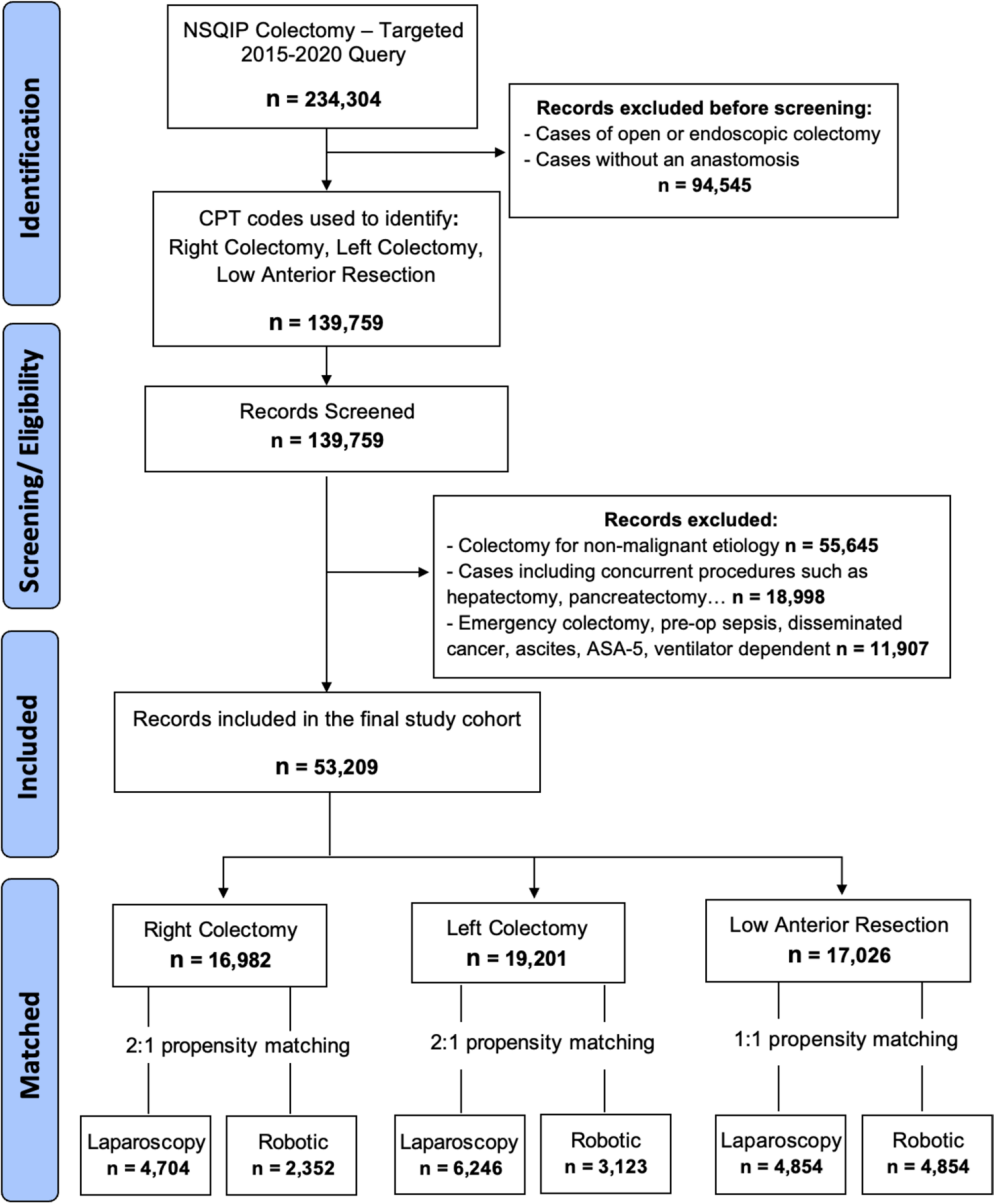
Study flow diagram showing inclusion and exclusion criteria

This is a retrospective cohort study using the ACS-NSQIP database from 2015 to 2020. Patients were identified using to the current procedural terminology (CPT) codes for colorectal procedures. Elective robotic or laparoscopic resections with anastomosis for CRC were included. In an effort to homogenize the study population, we serially excluded cases with disseminated cancer, ascites, preoperative sepsis, ASA-5, ventilator dependence, and concurrent major procedures such as hepatectomy or pancreatectomy. Cases were stratified according to the location of the colon or rectal resection: right-sided colectomy (CPT codes 44160, 44205), left-sided colectomy (44140, 44204), or low anterior resection (44207, 44208, 44145, 44146). The data for each of these three groups are presented separately. Using the “COL_APPROACH” variable, patients were divided into robotic or laparoscopic groups. Patients who had an unplanned conversion to open surgery remained in their original group (intention-to-treat). Lastly, we performed a subgroup analysis on patients undergoing LAR evaluating those who underwent a diverting loop ileostomy (CPT 44208, 44146), and those who did not (CPT 44207, 44145). Figure 1 depicts the study flow diagram with inclusion and exclusion criteria. This study was reported in accordance with the “Strengthening the Reporting of Observational Studies in Epidemiology” (STROBE) 2021 guidelines [22].

### Outcomes evaluated

We compared baseline preoperative characteristics of patients undergoing robotic and laparoscopic colorectal resections such as age, gender, race, body mass index (BMI), ASA class, and bowel preparation (mechanical and antibiotic). We assessed intraoperative outcomes: number of lymph nodes harvested, unplanned conversion to open, and operative time. Finally, we evaluated postoperative outcomes: length of hospital stay (LOS), textbook outcome, anastomotic leak, postoperative ileus, 30-day readmissions, complications, and mortality.

Relying on a single outcome with low event rates may not accurately reflect the perioperative course, thereby creating a need for a multidimensional indicator to incite improvement in quality of care. Textbook outcome (TO) is a novel surgical quality assessment tool that combines structure, process, and surgical outcome. It is a simple, useful, and reliable measure that has been validated in different surgical specialties, showing adequate discriminant validity [23, 24]. This composite quality metric incorporates several parameters, and many aspects of morbidity (complications, LOS, interventions, and readmission) to accurately reflect the perioperative course and most desirable outcome [25]. We defined TO as a length of hospital stay less than 5 days (75th percentile) and the absence of 30-day complications, readmission, or mortality. In line with previous literature adapting the Clavien-Dindo classification to the ACS-NSQIP, major morbidity was defined as any of the complications listed in Appendix Table 6 [26, 27].

### Statistical analysis

Statistical analyses were performed using R statistical software and the IBM SPSS statistical package (Version 28). After defining two treatment groups as robotic and laparoscopic, we performed propensity score matching (PSM) using the “MatchIt” and “optmatch” packages in R. We estimated the conditional probability of undergoing a robotic colorectal resection (the propensity score) using a multivariable logistic regression model. Next, we created balanced cohorts using 2-to-1 (laparoscopic to robotic) optimal pair matching for RC and LC, and 1-to-1 for LAR due to the higher number of robotic LARs. The choice of covariates included in the PSM was done according to the recommendations provided by Kainz et al. [28]. We also included covariates that were statistically significant on multivariate analysis. The PSM was done without replacement and with a “tol” argument of 10^–8^ dictating the numerical tolerance that determines when the optimal solution is found. Using standardized mean differences (SMD), we conducted balance diagnostics with SMD < 0.1 indicating a good balance and implying a negligible difference between treatment groups. Continuous variables with normal distribution are presented as mean and standard deviation (SD), while those with non-normal distributions are presented as median and interquartile range [IQR]. In the unmatched cohorts, we compared the baseline demographic and pathologic characteristics between the two groups with a chi-squared test for categorical variables. In the matched cohorts, considering the paired nature of the data, we used a McNemar test or McNemar-Bowker test for categorical variables and a Wilcoxon signed-rank test for continuous variables. Two-sided *p* values are reported. An *α* < 0.05 was considered statistically significant for all hypothesis testing.

## Results

### Patient characteristics and propensity score matching

**Fig. 2:**
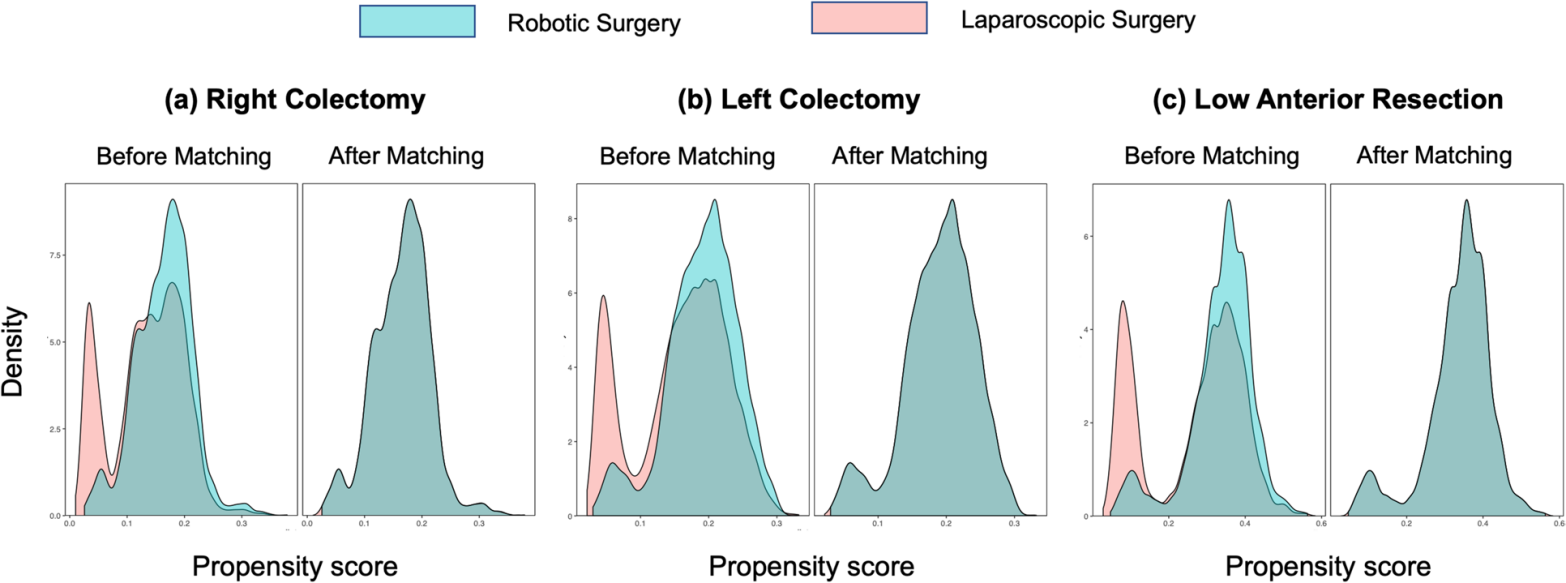
Density plots of propensity scores before and after optimal pair matching

**Fig. 3:**
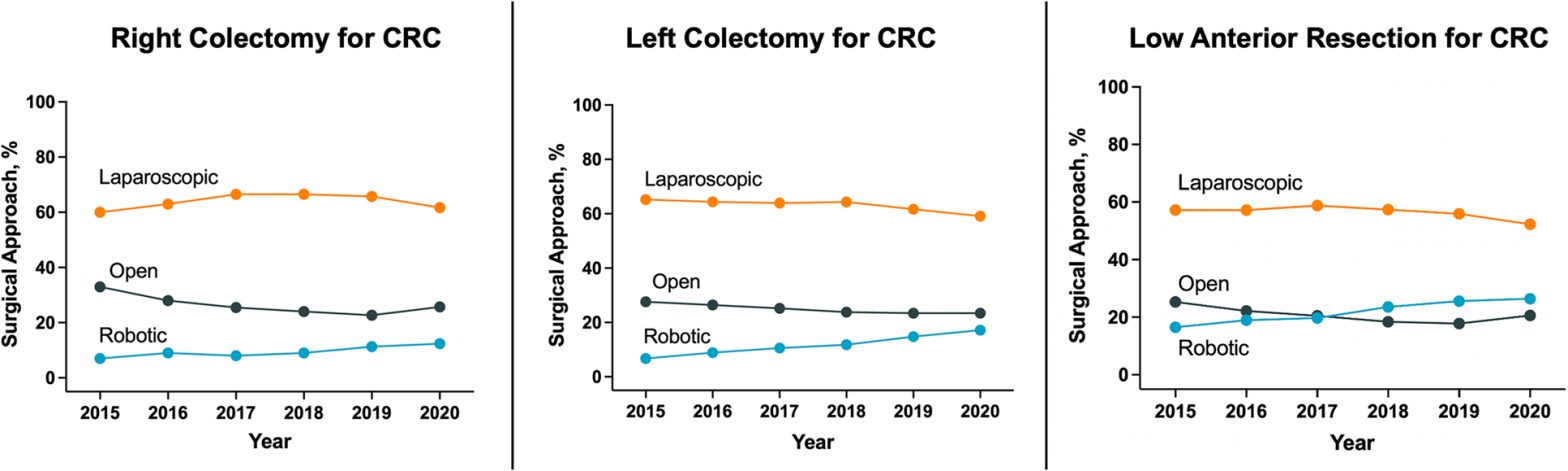
Trends in the surgical approach of colorectal cancer (CRC) in patients from the ACS-NSQIP (2015–2020)

We identified 234,304 patients in the colectomy targeted ACS-NSQIP (2015–2020). After screening for eligibility, 53,209 patients were included in the analysis: 16,982 had a RC, 19,201 LC, and 17,026 LAR. Figure 1 illustrates the distribution and matching results of patients stratified according to the location of their colorectal resection. Characteristics of patients included in the study cohort are described before and after matching in Tables 1, 2, and 3. For each of the three groups, the distribution of baseline covariates was adequately balanced in the matched data sets with the largest SMD = 0.048, implying a negligible discrepancy between treatment groups. Density plots of the matched data sets (Fig. 2) are nearly indistinguishable, implying a good balance of covariates based on the estimated propensity score. Figure 3 depicts the trends in the surgical approach of colorectal cancer (CRC) during our study period in patients from the ACS-NSQIP (2015–2020).

**Table 1:** Demographics and pathologic characteristics of patients undergoing right colectomy before and after propensity score matching

	**Unmatched right colectomy dataset**				**2:1 Matched right colectomy dataset**			
	Laparoscopic	Robotic	SMD	*p*§	Laparoscopic	Robotic	SMD	*p***‡**
**Sample size**	14,630	2352			4704	2352		
**Age, years**				< 0.01*				0.22
18–40	312 (2.1)	40 (1.7)	0.032		62 (1.3)	40 (1.7)	0.031	
41–60	3053 (20.9)	588 (25.0)	0.098		1130 (24.0)	588 (25.0)	0.023	
61–80	8716 (59.6)	1411 (60.0)	0.008		2860 (60.8)	1411 (60.0)	0.017	
> 80	2549 (17.4)	313 (13.3)	0.114		652 (13.9)	313 (13.3)	0.016	
**Sex**				0.87				0.62
Female	7723 (52.8)	1246 (53)	0.004		2517 (53.5)	1246 (53.0)	0.011	
Male	6907 (47.2)	1106 (47.0)	0.004		2187 (46.5)	1106 (47.0)	0.011	
**Race/ethnicity**				< 0.01*				0.17
White	9486 (64.8)	1839 (78.2)	0.299		3757 (79.9)	1839 (78.2)	0.041	
Black or AA	1516 (10.4)	282 (12.0)	0.052		556 (11.8)	282 (12.0)	0.005	
Asian	378 (2.6)	89 (3.8)	0.068		150 (3.2)	89 (3.8)	0.032	
Other	3250 (22.2)	142 (6.0)	0.477		241 (5.1)	142 (6.0)	0.040	
Hispanic	586 (4.0)	136 (5.8)	0.082		232 (4.9)	136 (5.8)	0.038	
**BMI**				< 0.01*				0.93
< 18	296 (2.0)	27 (1.1)	0.070		48 (1.0)	27 (1.1)	0.012	
18–25	4023 (27.5)	590 (25.1)	0.055		1202 (25.6)	590 (25.1)	0.011	
25–30	5026 (34.4)	766 (32.6)	0.038		1546 (32.9)	766 (32.6)	0.006	
> 30	5285 (36.1)	969 (41.2)	0.104		1908 (40.6)	969 (41.2)	0.013	
**ASA classification**				< 0.01*				0.74
Class 1	165 (1.1)	11 (0.5)	0.074		26 (0.6)	11 (0.5)	0.012	
Class 2	4818 (32.9)	786 (33.4)	0.010		1572 (33.4)	786 (33.4)	0.001	
Class 3	8549 (58.4)	1404 (59.7)	0.026		2829 (60.1)	1404 (59.7)	0.009	
Class 4	1098 (7.5)	151 (6.4)	0.043		277 (5.9)	151 (6.4)	0.022	
**Pathologic T stage**				< 0.01*				0.91
T1	1606 (11.0)	271 (11.5)	0.017		544 (11.6)	271 (11.5)	0.001	
T2	2417 (16.5)	434 (18.5)	0.051		835 (17.8)	434 (18.5)	0.018	
T3	7092 (48.5)	1091 (46.4)	0.042		2225 (47.3)	1091 (46.4)	0.018	
T4	1986 (13.6)	278 (11.8)	0.053		579 (12.3)	278 (11.8)	0.015	
Other	1529 (10.5)	278 (11.8)	0.044		521 (11.1)	278 (11.8)	0.023	
**Mechanical bowel prep**				< 0.01*				0.96
Yes	9018 (61.6)	1747 (74.3)	0.273		3497 (74.3)	1747 (74.3)	0.001	
No	5612 (38.4)	605 (25.7)	0.273		1207 (25.7)	605 (25.7)	0.001	
**Antibiotic bowel prep**				< 0.01*				0.53
Yes	7967 (54.5)	1596 (67.9)	0.278		3163 (67.2)	1596 (67.9)	0.013	
No	6663 (45.5)	756 (32.1)	0.278		1541 (32.8)	756 (32.1)	0.013	

Data are expressed as *n* (%) unless otherwise specified

*RC* Right colectomy, *SMD* Standardized mean difference, *ASA* American Society of Anesthesiology

^§^Indicates *p* value for *χ*^2^ test

^‡^Indicates *p* value for McNemar or McNemar-Bowker test

^*^Indicates statistical significance with *α* < 0.05

**Table 2:** Demographics and pathologic characteristics of patients undergoing left colectomy before and after propensity score matching

	**Unmatched left colectomy dataset**				**2:1 Matched left colectomy dataset**			
	Laparoscopic	Robotic	SMD	*p*§	Laparoscopic	Robotic	SMD	*p***‡**
**Sample size**	16,078	3123			6246	3123		
**Age, years**				< 0.01*				0.33
18–40	418 (2.6)	101 (3.2)	0.038		162 (2.6)	101 (3.2)	0.038	
41–60	4375 (27.2)	1022 (32.7)	0.121		2001 (32.0)	1022 (32.7)	0.015	
61–80	9005 (56.0)	1663 (53.3)	0.055		3392 (54.3)	1663 (53.3)	0.021	
> 80	2280 (14.2)	337 (10.8)	0.103		691 (11.1)	337 (10.8)	0.009	
**Sex**				0.014*				0.99
Female	8059 (50.1)	1490 (47.7)	0.048		2980 (47.7)	1490 (47.7)	0.001	
Male	8019 (49.9)	1633 (52.3)	0.048		3266 (52.3)	1633 (52.3)	0.001	
**Race/ethnicity**				< 0.01*				0.15
White	9860 (61.3)	2385 (76.4)	0.329		4868 (77.9)	2385 (76.4)	0.037	
Black or AA	1532 (9.5)	382 (12.2)	0.087		701 (11.2)	382 (12.2)	0.031	
Asian	1212 (7.5)	170 (5.4)	0.085		314 (5.0)	170 (5.4)	0.019	
Other	3474 (21.6)	186 (6.0)	0.466		363 (5.8)	186 (6.0)	0.006	
Hispanic	817 (5.1)	174 (5.6)	0.022		314 (5.0)	174 (5.6)	0.024	
**BMI**				< 0.01*				0.73
< 18	332 (2.1)	42 (1.3)	0.056		75 (1.2)	42 (1.3)	0.013	
18–25	4452 (27.7)	784 (25.1)	0.059		1580 (25.3)	784 (25.1)	0.004	
25–30	5602 (34.8)	1022 (32.7)	0.045		2075 (33.2)	1022 (32.7)	0.011	
> 30	5692 (35.4)	1275 (40.8)	0.112		2516 (40.3)	1275 (40.8)	0.011	
**ASA classification**				0.021*				0.02*
Class 1	282 (1.8)	42 (1.3)	0.033		67 (1.1)	42 (1.3)	0.025	
Class 2	5886 (36.6)	1148 (36.8)	0.003		2312 (37.0)	1148 (36.8)	0.005	
Class 3	8939 (55.6)	1781 (57.0)	0.029		3624 (58.0)	1781 (57.0)	0.020	
Class 4	971 (6.0)	152 (4.9)	0.052		243 (3.9)	152 (4.9)	0.048	
**Pathologic T stage**				< 0.01*				0.91
T1	1898 (11.8)	418 (13.4)	0.048		854 (13.7)	418 (13.4)	0.008	
T2	2565 (16.0)	524 (16.8)	0.022		1052 (16.8)	524 (16.8)	0.002	
T3	7723 (48.0)	1417 (45.4)	0.053		2859 (45.8)	1417 (45.4)	0.008	
T4	1901 (11.8)	329 (10.5)	0.041		633 (10.1)	329 (10.5)	0.013	
Other	1991 (12.4)	435 (13.9)	0.046		848 (13.6)	435 (13.9)	0.010	
**Mechanical bowel prep**				< 0.01*				0.71
Yes	10,295 (64.0)	2277 (72.9)	0.192		4572 (73.2)	2277 (72.9)	0.006	
No	5783 (36.0)	847 (27.1)	0.192		1674 (26.8)	846 (27.1)	0.006	
**Antibiotic bowel prep**				< 0.01*				0.91
Yes	8121 (50.5)	1940 (62.1)	0.236		3886 (62.2)	1940 (62.1)	0.002	
No	7957 (49.5)	1183 (37.9)	0.236		2360 (37.8)	1183 (37.9)	0.002	

Data are expressed as *n* (%) unless otherwise specified

*LC* Left colectomy, *SMD* Standardized mean difference, *ASA* American Society of Anesthesiology

^§^Indicates *p* value for *χ*^2^ test

^‡^Indicates *p* value for McNemar or McNemar-Bowker test

^*^Indicates statistical significance with *α* < 0.05

**Table 3:** Demographics and pathologic characteristics of patients undergoing low anterior resection before and after propensity score matching

	**Unmatched low anterior resection dataset**				**1:1 Matched low anterior resection dataset**			
	Laparoscopic	Robotic	SMD	*p*§	Laparoscopic	Robotic	SMD	*p***‡**
**Sample size**	12,172	4854			4854	4854		
**Age, years**				< 0.01*				0.52
18–40	559 (4.6)	250 (5.2)	0.026		227 (4.7)	250 (5.2)	0.022	
41–60	5106 (41.9)	2318 (47.8)	0.117		2353 (48.5)	2318 (47.8)	0.014	
61–80	5682 (46.7)	2059 (42.4)	0.086		2062 (42.5)	2059 (42.4)	0.001	
> 80	825 (6.8)	227 (4.7)	0.091		212 (4.4)	227 (4.7)	0.015	
**Sex**				< 0.01*				0.31
Female	5136 (42.2)	1905 (39.2)	0.060		1954 (40.3)	1905 (39.2)	0.021	
Male	7036 (57.8)	2949 (60.8)	0.060		2900 (59.7)	2949 (60.8)	0.021	
**Race/ethnicity**				< 0.01*				0.18
White	7018 (57.7)	3799 (78.3)	0.453		3869 (79.7)	3799 (78.3)	0.035	
Black or AA	724 (5.9)	329 (6.8)	0.034		312 (6.4)	329 (6.8)	0.014	
Asian	705 (5.8)	346 (7.1)	0.054		303 (6.2)	346 (7.1)	0.035	
Other	3725 (30.6)	380 (7.8)	0.604		370 (7.6)	380 (7.8)	0.008	
Hispanic	654 (5.4)	331 (6.8)	0.060		316 (6.5)	331 (6.8)	0.012	
**BMI**				< 0.01*				0.13
< 18	211 (1.7)	65 (1.3)	0.032		46 (0.9)	65 (1.3)	0.037	
18–25	3500 (28.8)	1319 (27.2)	0.035		1274 (26.2)	1319 (27.2)	0.021	
25–30	4322 (35.5)	1664 (34.3)	0.026		1665 (34.3)	1664 (34.3)	0.001	
> 30	4139 (34.0)	1806 (37.2)	0.067		1869 (38.5)	1806 (37.2)	0.027	
**ASA Classification**				< 0.01*				0.58
Class 1	302 (2.5)	63 (1.3)	0.087		49 (1.0)	63 (1.3)	0.027	
Class 2	5263 (43.2)	2135 (44.0)	0.015		2142 (44.1)	2135 (44.0)	0.003	
Class 3	6105 (50.2)	2535 (52.2)	0.041		2563 (52.8)	2535 (52.2)	0.012	
Class 4	502 (4.1)	121 (2.5)	0.091		100 (2.1)	121 (2.5)	0.029	
**Pathologic T stage**				< 0.01*				0.69
T1	1406 (11.6)	561 (11.6)	0.001		574 (11.8)	561 (11.6)	0.008	
T2	2460 (20.2)	1139 (23.5)	0.079		1096 (22.6)	1139 (23.5)	0.021	
T3	5395 (44.3)	2027 (41.8)	0.052		2072 (42.7)	2027 (41.8)	0.019	
T4	943 (7.7)	260 (5.4)	0.097		265 (5.5)	260 (5.4)	0.005	
Other	1968 (16.2)	867 (17.9)	0.045		847 (17.4)	867 (17.9)	0.011	
**Mechanical bowel prep**				0.74				0.31
Yes	9093 (74.7)	3638 (74.9)	0.006		3681 (75.8)	3638 (74.9)	0.021	
No	3079 (25.3)	1216 (25.1)	0.006		1173 (24.2)	1216 (25.1)	0.021	
**Antibiotic bowel prep**				< 0.01*				0.75
Yes	7097 (58.3)	3389 (69.8)	0.242		3375 (69.5)	3389 (69.8)	0.006	
No	5075 (41.7)	1465 (30.2)	0.242		1479 (30.5)	1465 (30.2)	0.006	

Data are expressed as *n* (%) unless otherwise specified

*LAR* Low anterior resection, *SMD* Standardized mean difference, *ASA* American Society of Anesthesiology

^§^Indicates *p* value for *χ*^2^ test

^‡^Indicates *p* value for McNemar or McNemar-Bowker test

^*^Indicates statistical significance with *α* < 0.05

### Right and left colonic resections

**Fig. 4:**
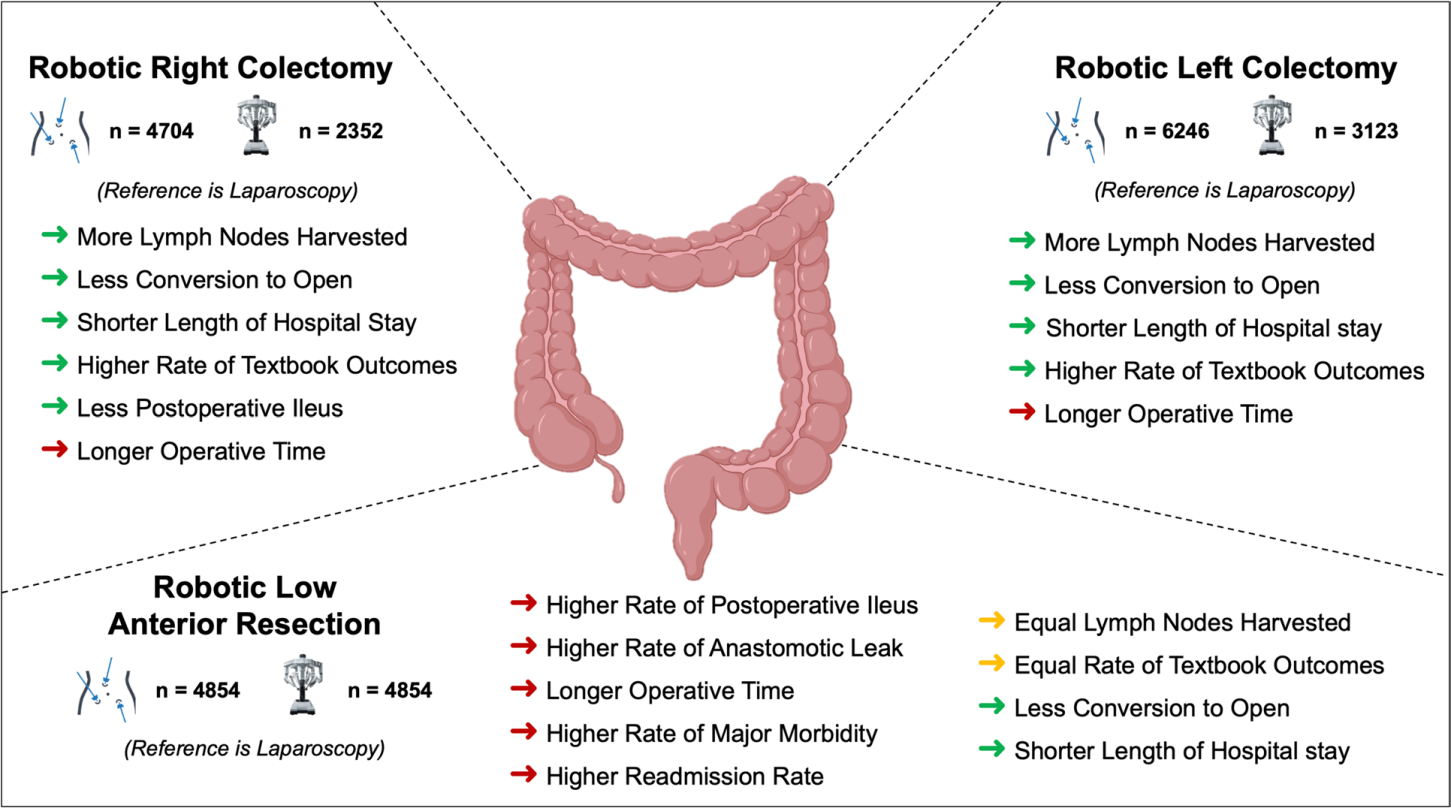
Summary of perioperative outcomes of right colectomy, left colectomy, and low anterior resection after propensity score matching

Tables 1 and 2 illustrate the characteristics of patients undergoing RC and LC, respectively. Each robotic case was matched to two laparoscopic. Before matching, most variables had a statistically significant difference (*p* < 0.05) between the two groups. Subsequently, after performing the PSM, all variables were homogenously balanced (SMD < 0.1). Baseline demographics of the unmatched cohorts revealed that patients undergoing robotic RC and LC for CRC were more likely to be young, white, obese, and receive mechanical or antibiotic bowel prep (Tables 1 and 2). For all perioperative outcomes, we evaluated the 2:1 laparoscopic to robotic matched data sets (Table 4). Figure 4 illustrates the perioperative outcomes of robotic surgery for CRC compared to laparoscopy.

**Table 4:** Perioperative outcomes of right and left colectomy after propensity score matching

	**2:1 Matched right colectomy dataset**			**2:1 Matched left colectomy dataset**		
	Laparoscopic Right colectomy	Robotic Right colectomy	*P* value	Laparoscopic Left colectomy	Robotic Left colectomy	*P* value
Sample size	4704	2352		6246	3123	
Operative time, median [IQR], minutes	134 [104–176]	183 [146–229]	< 0.001*	154 [117–206]	202 [160–259]	< 0.001*
Lymph nodes evaluated, mean (SD)	22.57 (10)	23.84 (11)	< 0.001*	21.03 (10)	21.70 (11)	< 0.001*
Bleeding transfusion occurence	392 (8.3)	188 (8.0)	0.569	382 (6.1)	179 (5.7)	0.385
Conversion to open	399 (8.5)	97 (4.1)	< 0.001*	551 (8.8)	161 (5.2)	< 0.001*
Postoperative ileus	546 (11.6)	211 (9.0)	< 0.001*	539 (8.6)	248 (7.9)	0.170
Anastomotic leak	87 (1.8)	44 (1.9)	1	113 (1.8)	65 (2.1)	0.301
Textbook outcome	3012 (64)	1672 (71)	< 0.001*	4254 (68.1)	2331 (74.6)	< 0.001*
Any complication	779 (16.6)	375 (15.9)	0.428	870 (13.9)	398 (12.7)	0.055
Major morbidity (> 2 Clavien-Dindo grade)	296 (6.3)	154 (6.5)	0.638	358 (5.7)	173 (5.5)	0.671
Length of hospital stay, mean (SD), days	4.7 (3.8)	4.0 (3.4)	< 0.001*	4.6 (3.8)	4.0 (3.5)	< 0.001*
30-day readmission	396 (8.4)	197 (8.4)	0.970	435 (7)	223 (7.1)	0.729
30-day mortality	45 (1)	16 (0.7)	0.171	48 (0.80)	29 (0.9)	0.378

Data are expressed as *n* (%) unless otherwise specified

*IQR* Interquartile range, *SD* Standard deviation

^*^Indicates statistical significance with *α* < 0.05

All results are reported as robotic vs. laparoscopic unless otherwise specified. When addressing intraoperative outcomes, the median operative time was longer in robotic compared to laparoscopic resections (183 vs. 134 min for RC, 202 vs. 154 for LC; *p* < 0.001). The average number of lymph nodes (LN) harvested during the operation, as documented in the pathology report, was higher in the robotic group (23.84 vs. 22.57 LN for RC, 21.70 vs. 21.03 for LC; *p* < 0.001). Robotic resection was associated with a lower conversion rate compared to laparoscopy (4.1% vs. 8.5% for RC, 5.2% vs. 8.8% for LC; *p* < 0.001). Finally, the number of bleeding transfusion occurrences within 72 h of operative start time was similar in the two groups (8.0% vs. 8.3% for RC; *p* = 0.57 and 5.7% vs. 6.1% for LC; *p* = 0.39).

When comparing postoperative outcomes, robotic and laparoscopic resections have comparable rates of anastomotic leak (1.9% vs. 1.8% for RC; *p* = 1 and 2.1% vs. 1.8% for LC; *p* = 0.301). The rate of postoperative ileus was significantly lower only in robotic RC (9.0% vs. 11.6%; *p* < 0.001), while it was comparable for both surgical approaches in LC (7.9% vs. 8.6%; *p* = 0.170). Both operative approaches had comparable overall complication rates (15.9% vs. 16.6% for RC; *p* = 0.43 and 12.7% vs. 13.9% for LC; *p* = 0.055), major morbidity (6.5% vs. 6.3% for RC; *p* = 0.64 and 5.5% vs. 5.7% for LC; *p* = 0.67), and 30-day mortality (0.7% vs. 1% for RC; *p* = 0.17 and 0.9% vs. 0.8% for LC; *p* = 0.38) (Table 4). Finally, robotic RC and LC were associated with a higher rate of textbook outcomes compared to laparoscopy (71.0% vs. 64.0% for RC and 74.6% vs. 68.1% for LC; *p* < 0.001). The apparent significant difference in textbook outcomes was driven by the shorter LOS and the lower rate of any complications for both RC and LC. Although complication rates are not lower in the robotic group on univariate analysis, they are contributing to the higher rates of TO.

### Low anterior resection

A total of 4854 patients undergoing robotic LAR were matched 1:1 to laparoscopic cases. Characteristics of the patient cohort undergoing LAR for CRC are illustrated in Table 3. Baseline demographics of the unmatched cohort revealed that patients undergoing robotic LAR were more likely to be young, white, obese, and receive mechanical or antibiotic bowel prep (Table 3). For all perioperative outcomes discussed below, we evaluated the 1:1 laparoscopic to robotic propensity score-matched data set (Table 5).

**Table 5:** Perioperative outcomes of low anterior resection after propensity score matching

	**1:1 Matched low anterior resection dataset**		
	Laparoscopic LAR	Robotic LAR	*P* value
Sample size	4854	4854	
Operative time, median [IQR], minutes	201 [151–264.25]	246 [193–320]	< 0.001*
Lymph nodes evaluated, mean (SD)	20.28 (10)	20.14 (10)	0.744
Bleeding transfusion occurence	143 (2.9)	121 (2.5)	0.189
Conversion to open	503 (10.4)	191 (3.9)	< 0.001*
Postoperative ileus	511 (10.5)	579 (11.9)	0.032*
Anastomotic leak	117 (2.4)	165 (3.4)	0.005*
Textbook outcome	3254 (67)	3303 (68)	0.297
Any complication	580 (11.9)	591 (12.2)	0.754
Major morbidity (> 2 Clavien-Dindo grade)	283 (5.8)	345 (7.1)	0.012*
Length of hospital stay, mean (SD), days	4.8 (4)	4.7 (3.8)	< 0.001*
30-day readmission	440 (9.1)	507 (10.4)	0.023*
30-day mortality	20 (0.4)	18 (0.4)	0.871

Data are expressed as *n* (%) unless otherwise specified

*IQR* Interquartile range, *SD* Standard deviation

^*^Indicates statistical significance with *α* < 0.05

When comparing intraoperative outcomes, the median operative time was longer in robotic LAR (246 vs. 201 min; *p* < 0.001). The average number of lymph nodes (LN) harvested was comparable in the two groups (20.14 vs. 20.28 LN; *p* = 0.744). Robotic resection was associated with a lower conversion rate (3.9% vs. 10.4%; *p* < 0.001). Finally, the number of bleeding transfusion occurrences was similar in the two groups (2.5% vs. 2.9%; *p* = 0.189).

When comparing postoperative outcomes, the robotic approach was associated with a higher rate of anastomotic leak compared to laparoscopy (3.4% vs. 2.4%; *p* = 0.005). Similarly, the rate of postoperative ileus was significantly higher in robotic LAR (11.9% vs. 10.5%; *p* = 0.032). Both operative approaches had comparable overall complication rates (12.2% vs. 11.9%; *p* = 0.754), but robotic LAR was associated with a higher rate of major morbidity (7.1% vs 5.8%; *p* = 0.012). Finally, the two surgical approaches had comparable rates of textbook outcomes (68% vs 67%; *p* = 0.297) and 30-day mortality (0.4% vs 0.4%; *p* = 0.871; Table 5).

Similarly, a subgroup analysis comparing patients undergoing LAR without a diverting loop ileostomy showed a higher rate of anastomotic leaks, major morbidity, and readmission with the robotic approach (Appendix Table 7). However, when evaluating patients undergoing LAR with a diverting loop ileostomy, both the robotic and laparoscopic approaches had a comparable rate of perioperative morbidity (Appendix Table 8).

## Discussion

In the USA, colorectal resections are among the most commonly performed surgical procedures and robotic surgery is being increasingly adopted in the management of CRC. Evidence supporting this transition from traditional laparoscopy has not been sufficient. To the best of our knowledge, this study is the largest retrospective propensity score-matched analysis comparing perioperative outcomes of robotic and laparoscopic resections for CRC. Our results suggest an advantage for the robotic approach in RC and LC by increasing the rate of textbook outcomes, decreasing conversion rate, and comparable morbidity and mortality. Conversely, robotic LAR was associated with a similar rate of TO compared to laparoscopy and an increased rate of postoperative ileus, anastomotic leak, and major morbidity.

In recent years, an increasing number of studies investigated the perioperative outcomes of minimally invasive surgery using the NSQIP database [29, 30]. El Aziz et al. report a comparative study highlighting the increased adoption of robotic colorectal surgery and its implications on perioperative outcomes [29]. They compared open, laparoscopic, and robotic colectomies performed for any etiology combining left, right, and low anterior resections. Similarly, a recent study by Soliman et al. also compared the two approaches for CRC and chronic diverticulitis using the NSQIP database [31]. Although some of the endpoints examined in these two studies are identical to our outcomes, we believe that stratifying resections by their location and performing a propensity score-matched analysis extend a deeper understanding of the data, and may uncover new insights that traditional statistical approaches cannot. When compared to rectal resections, RC and LC have fundamentally different technical and perioperative considerations; thus, it is essential to investigate each of these populations separately. Additionally, although the NSQIP provides colectomy data for various etiologies, our study compared the two surgical approaches in the management of CRC only.

A systematic review and meta-analysis by Tschann et al. showed more favorable perioperative outcomes with robotic RC compared to laparoscopy such as a lower rate of blood loss, lower conversion rate, and shorter LOS [32]. Another systematic review and meta-analysis by Solaini et al. concluded that robotic RC is non-inferior to laparoscopy in terms of postoperative complications and mortality [33]. Our study analogously demonstrates several advantages of robotic RC such as a higher rate of textbook outcomes, shorter LOS, lower conversion rate, and less postoperative ileus. With only one randomized controlled trial included, the main limitation of these two systematic reviews was that most included studies were retrospective, potentially contributing to a selection bias. Although our study is also retrospective, we performed a PSM analysis to mitigate the impact of selection bias.

In a recent systematic review and meta-analysis of patients undergoing LC, Solaini et al. concluded that the robotic approach is associated with lower postoperative complications and morbidity [15]. Interestingly, their results were not confirmed in the subgroup analysis done for malignant etiologies. Operative time was longer in the robotic group, while conversion rate was lower. This is in line with the findings of our study, which demonstrated comparable perioperative complication and mortality rates in LC for CRC. Our study extends a deeper understanding of this comparison and highlights the increased rate of textbook outcomes with robotic LC. We postulate that the robotic approach may be improving outcomes in RC and LC due to better 3D visualization, greater degrees-of-freedom, and the ability to precisely perform complex maneuvers in narrow anatomical spaces compared to laparoscopy.

Robotic LAR is a more complex and intricate procedure compared to RC and LC with an estimated learning curve of 55–65 cases, compared to 35–45 for LC, and 16–25 for RC [34, 35]. Using a national clinical database, Matsuyama et al. recently compared the perioperative outcomes of robotic and laparoscopic LAR in a propensity score-matched analysis in patients with rectal cancer [36]. They showed improved perioperative outcomes with robotic LAR such as a lower conversion rate, a shorter LOS, comparable complication rates, and a lower mortality rate. Furthermore, a meta-analysis by Sun et al. also showed a shorter LOS, lower conversion rate, and lower overall complication rate with robotic LAR compared to laparoscopy [37]. Although our study demonstrated a shorter LOS after robotic LAR and lower a conversion rate, it challenges some of the findings proposed by the two aforementioned studies. Our results showed a higher rate of severe complications and an increase in leak rates, postoperative ileus, and 30-day readmission after robotic LAR compared to laparoscopy. The higher leak rates with robotic LAR may be due to the long learning curve of this procedure or due to a selection bias for lower-lying rectal tumors being done robotically to use the advantages of this technology in the narrow pelvis. Contrary to our initial hypothesis, the results of this study did not substantiate our expected outcomes for low anterior resection. Instead, the data suggest a higher rate of postoperative morbidity with robotic LAR and no significant difference in the rate of textbook outcomes. This can be partially attributed to the fact that TO only considers overall (any) complications and not severe complications. Additionally, although the LOS demonstrated a statistically significant advantage in favor of the robotic approach, the actual difference was only 0.1 days, which may not have a clinically relevant impact.

In the ROLARR randomized controlled trial, Jayne et al. compared the conversion rate of robotic and laparoscopic rectal resection in 471 patients between 2011 and 2014 [38]. They reported a conversion rate of 8.1% for robotic and 12.2% for laparoscopic, but this difference was not statistically significant. Interestingly, a sensitivity analysis exploring learning effects suggested a potentially lower robotic conversion rate when performed by surgeons with substantial prior robotic experience. Our study indicates that the conversion rate for laparoscopic LAR was 10.4% during the study period which was significantly higher that the robotic conversion rate (3.9%). In a recent multicenter trial, Feng et al. reported better postoperative recovery with the robotic approach for middle and low rectal cancer (REAL trial) [39]. The strength of this trial lies in the selection of middle and low rectal cancer cases which are theoretically the narrow anatomical spaces where robotic surgery is expected to confer an advantage over laparoscopy. In our current study, the LAR group included low, middle, and high rectal cancer because the ACS-NSQIP does not provide data on the distance of the tumor from the anal verge to stratify them.

Our study has several important limitations that need to be addressed. First, despite being one of the best available tools for quality improvement in surgery, the NSQIP database carries inherent constraints. Errors in classification, coding, or reporting of patient information may affect the quality of the data. Additionally, the NSQIP only collects data from around 850 or 14% of all US hospitals, further increasing the risk of selection bias towards more developed and higher performing centers. However, the large sample size generated from this national database allowed for a robust statistical analysis, which increases the accuracy of results, particularly when comparing procedures with small, expected differences. Second, this was a retrospective cohort study which carries a risk of selection bias. Even after implementing a PSM, residual selection bias from unmeasured/unknown confounders cannot be excluded in the absence of randomization. Third, the NSQIP does not provide data on neoadjuvant radiotherapy for CRC, an already established risk factor for postoperative complication. Fourth, there was no consideration of surgeon expertise level, and the nuanced variations of case complexity were not captured and accounted for by the included variables. The database lacks granular data allowing us to stratify participating institutions into high-volume/low-volume centers, and it lacks any information on the distance of rectal tumors from the anal verge which contributes to the level of complexity of the case. Additionally, the expertise level of the surgeon performing the operation is unknown and their experience with either laparoscopic or robotic colectomy is not clearly defined. RC, LC, and LAR have different learning curves that must be evaluated in a multidimensional approach when comparing robotic and laparoscopic surgery [35]. Despite being a limitation of our study, the suspected heterogeneous expertise levels between different contributing centers reflects the current real-world practice, thus enhancing the external validity and generalizability of this study. Additionally, the NSQIP does not report technical aspects of the procedure such as an intracorporeal vs extracorporeal anastomosis, or the extent of lymphadenectomy (D2 vs D3), which are known to affect OR time and other perioperative outcomes. It should also be acknowledged that differences in short-term outcomes such as LOS may be mediated by differences in postoperative care pathways. Finally, the short follow-up period reported by the ACS-NSQIP (30 days post-op) limits our ability to assess long-term oncologic and survival outcomes.

## Conclusions

In this retrospective cohort study, robotic right and left colectomy for CRC showed an increase in textbook outcomes with a comparable morbidity and mortality compared to laparoscopy. Conversely, albeit limited by several possible confounders, low anterior resection showed increased rates of anastomotic leak, postoperative ileus, major morbidity, and a comparable rate of textbook outcomes. As robotic colorectal surgery comes with an increased fiscal burden, the enthusiasm accompanying it should not outpace the evidence needed to support its expansion. The associations highlighted in our study should be considered in the surgical planning for patients with colorectal cancer.

## Data Availability

The datasets generated during and/or analyzed during the current study are available from the corresponding author on reasonable request.

## Appendix A

**Table:** **Table 6** Serious complications causing major morbidity in the 30-day follow-up period

**Serious complication**	
Organ space infection	Anastomotic leak
Wound dehiscence	Deep venous thrombosis
Pulmonary embolism	Failure to wean off ventilator
Acute renal failure	Stroke or cardiovascular event
Cardiac arrest	Myocardial infarction
Sepsis or septic shock	

## Appendix B

**Table:** **Table 7** Perioperative outcomes of low anterior resection without diverting loop ileostomy

	**Low anterior resection** **without diverting loop ileostomy**		
	Laparoscopic LAR without ileostomy	Robotic LAR without ileostomy	*P* value
Sample size	4324	4034	
Operative time, median [IQR], minutes	197 [148 – 258]	239 [188 – 310]	< 0.001*
Lymph nodes evaluated, mean (SD)	20.5 (9.8)	20.6 (9.8)	0.950
Bleeding transfusion occurence	126 (2.9)	93 (2.3)	0.082
Conversion to open	426 (9.9)	139 (3.4)	< 0.001*
Postoperative ileus	413 (9.6)	411 (10.2)	0.329
Anastomotic leak	101 (2.3)	134 (3.3)	0.006*
Textbook outcome	2999 (69.4)	2867 (71.1)	0.087
Any complication	486 (11.2)	452 (11.2)	0.960
Major morbidity (> 2 Clavien-Dindo grade)	228 (5.3)	265 (6.6)	0.012*
Length of hospital stay, mean (SD), days	4.6 (3.9)	4.4 (3.7)	< 0.001*
30-day readmission	354 (8.2)	384 (9.5)	0.032*
30-day mortality	18 (0.4)	13 (0.3)	0.480

Data are expressed as *n* (%) unless otherwise specified

*IQR* Interquartile range, *SD* Standard deviation

^*^Indicates statistical significance with *α* < 0.05

## Appendix C

**Table:** **Table 8** Perioperative outcomes of low anterior resection with diverting loop ileostomy

	**Low anterior resection** **with diverting loop ileostomy**		
	Laparoscopic LAR + Ileostomy	Robotic LAR + Ileostomy	*P* value
Sample size	530	820	
Operative time, median [IQR], minutes	243 [185–328]	279 [221–348]	< 0.001*
Lymph nodes evaluated, mean (SD)	18.6 (10)	18.2 (8)	0.853
Bleeding transfusion occurence	17 (3.2)	28 (3.4)	0.878
Conversion to open	77 (14.5)	52 (6.3)	< 0.001*
Postoperative ileus	98 (18.5)	159 (19.4)	0.681
Anastomotic leak	16 (3.0)	31 (3.8)	0.456
Textbook outcome	255 (48)	436 (53)	0.069
Any complication	94 (17.7)	139 (17)	0.709
Major morbidity (> 2 Clavien-Dindo grade)	55 (10.4)	80 (9.8)	0.710
Length of hospital stay, mean (SD), days	6.6 (5)	5.9 (4.4)	0.004*
30-day readmission	86 (16.2)	123 (15)	0.543
30-day mortality	2 (0.4)	5 (0.6)	0.562

Data are expressed as *n* (%) unless otherwise specified

*IQR* Interquartile range, *SD* Standard deviation

^*^Indicates statistical significance with *α* < 0.05

## Abbreviations

*CRC*: Colorectal cancer
*ACGME*: Accreditation Council for Graduate Medical Education
*ACS-NSQIP*: American College of Surgeons-National Surgical Quality Improvement Program
*RC*: Right colectomy
*LC*: Left colectomy
*LAR*: Low anterior resection
*CPT*: Current procedural terminology
ASA: American Society of Anesthesiology
*BMI*: Body mass index
*LOS*: Length of hospital stay
*TO*: Textbook outcome
*PSM*: Propensity score matching
*SMD*: Standardized mean difference
*SD*: Standard deviation
*IQR*: Interquartile range
